# From Innovation to Implementation: Transforming Geriatric Care in the VA System

**DOI:** 10.1093/geroni/igaf122.480

**Published:** 2025-12-31

**Authors:** Caroline Madrigal, Lea Kiefer, Marianne Shaughnessy

## Abstract

Veterans Affairs (VA) has a long-standing commitment to providing high-quality geriatric care, driven by a history of innovation and a dedication to meeting the unique needs of older Veterans. This symposium will provide an overview of the evolution of geriatric clinical innovations in VA, highlighting organizational milestones, successful models of care, and strategies for sustainable implementation. We will review the development of VA’s geriatric care infrastructure, examining the establishment of Geriatrics and Extended Care and the Geriatric Research, Education, and Clinical Centers (GRECCs), and their impact on the research—clinical practice continuum. We will showcase specific examples of innovative, evidence-based geriatric care models developed in VA, supported by VA’s national GRECC office, and disseminated across the enterprise via GRECC’s Mentoring Partnerships Program. We will explore the origin, evidence-base, and strategies for long-term sustainment for each program—specifically: 1) Coordinated Transitional Care (CTraC)— a post-hospital transitional care program to support Veterans at high-risk of readmission; 2) Patient Priorities Care— an Age-friendly approach to align current care plans with patients’ unique health priorities; 3) STRIDE (AssiSTed EaRly MobIlity for HospitalizeD VEterans )— a supervised walking program designed to enhance older Veterans’ physical function during hospitalization; 4) Gerofit— an outpatient supervised group exercise program designed to improve physical function and maintain independence in older Veterans with multimorbidity. This symposium will offer valuable insights into VA’s journey in geriatric innovations, demonstrating advancements in care for older Veterans and providing a roadmap for continued progress in the field of geriatric care.

